# Improving diversity in study participation: Patient perspectives on barriers, racial differences and the role of communities

**DOI:** 10.1111/hex.13554

**Published:** 2022-06-28

**Authors:** Lisa Shea, Jacqueline Pesa, Gabrielle Geonnotti, Valerie Powell, Caryl Kahn, Wesley Peters

**Affiliations:** ^1^ Janssen Scientific Affairs, LLC Titusville New Jersey USA; ^2^ CorEvitas, LLC Waltham Massachusetts USA

**Keywords:** diversity, ethnicity, patient engagement, qualitative research, race, study participation

## Abstract

**Introduction:**

The lack of racial/ethnic diversity in research potentially limits the generalizability of findings to a broader population, highlighting the need for greater diversity and inclusion in clinical research. Qualitative research (i.e., focus groups) was conducted to identify (i) the potential motivators and barriers to study participation across different races and ethnicities; (ii) preferred delivery of education and information to support healthcare decision‐making and the role of the community.

**Methods:**

Patient focus groups were conducted with 26 participants from the sponsor's Patient Engagement Research Councils selected through subjective sampling. Recruitment prioritized adequate representation across different race/ethnic groups. Participation was voluntary and participants underwent a confidential interview process before selection. Narrative analysis was used to identify themes and draw insights from interactions. Experienced research specialists identified emerging concepts, and these were tested against new observations. The frequency of each concept was examined to understand its importance.

**Results:**

Based on self‐selected race/ethnicity, participants were divided into five focus groups (Groups: African American/Black: 2; Hispanic/Latino, Asian American, and white: 1 each) and were asked to share their experiences/opinions regarding the stated objectives. Barriers to study participation included: limited awareness of opportunities to participate in research, fears about changes in standard therapy, breaking cultural norms/stigma, religion‐related concerns and mistrust of clinical research. Participants identified the importance of transparency by pharmaceutical companies and other entities to build trust and partnership and cited key roles that communities can play. The perceptions of the African American group regarding diversity/inclusion in research studies appeared to be different from other groups; a lack of trust in healthcare providers, concerns about historical instances of research abuse and the importance of prayer were cited.

**Conclusion:**

This study provided insights into barriers to study participation, and also highlighted the need for pharmaceutical companies and other entities to authentically engage in strategies that build trust within communities to enhance recruitment among diverse populations.

**Patient or Public Contribution:**

The data collected in the present study was provided by the participants in the focus groups.

## 1 INTRODUCTION

Carefully designed randomized clinical trials, along with complementary findings from observational and real‐world evidence studies, can demonstrate the efficacy, safety and treatment outcomes of therapies in a certain patient population.^1^, ^2^, ^3^ Ideally, participants in research studies should closely mimic the demographic diversity of the general population or the prevalence of a specific disease.^4^, ^5^, ^6^, ^7^ The responses to a particular medication might differ among population subgroups based on several factors, such as age, sex, genetic profile and ethnicity.^8^, ^9^, ^10^ Although race and ethnicity are interchangeably used, ‘race’ refers to a person's physical characteristics, and ‘ethnicity’ includes culture, beliefs and language in addition to the racial ancestry. A single geographical area can have few basic races, but a number of ethnic groups.^8^ Genetic variability in ancestral populations, along with shared environmental factors, is known to alter the response to drug therapy among different ethnic subgroups.^9^ Therefore, the inclusion of different races and ethnicities in research is critical to enable the development of better treatments and better ways to fight diseases that often disproportionately impact diverse communities.

Recent studies have demonstrated that study samples do not adequately represent the race and ethnicity of those affected by various diseases.^7^, ^11^, ^12^, ^13^ For instance, more than 40% of cancer clinical trials in the United States do not reflect the incidence rates among diverse racial/ethnic groups, resulting in an over‐representation of whites.^11^ Under‐representation of non‐white participants has been widely reported in US‐based clinical and observational studies, including those for type I diabetes,^14^ type II diabetes,^15^ Duchenne muscular dystrophy^16^ and rheumatoid arthritis.^17^ Importantly, the 2020 Census reported a decrease in the white alone population by 8.6%, a 276% increase in the multiracial (two or more races) population and 23% and 88.7% growth in the Hispanic/Latino and Black/African American populations, respectively, when compared with the 2010 Census.^18^ By 2045, it is projected that less than 50% of the US population will be comprised of non‐Hispanic whites.^19^

Study findings with homogeneous samples (e.g., those based on age, gender, or race/ethnicity) are less generalizable to the broader population.^7^, ^20^ While medical care and scientific knowledge have advanced significantly over the past decade, many studies continue to have a majority of white participants.^21^ African American, Latino and other diverse racial/ethnic groups are often less represented in these studies and can have worse health outcomes as a result.^7^, ^22^, ^23^ Studies featuring an adequate representation of diverse racial/ethnic groups therefore are critical to reducing healthcare disparities.^24^, ^25^, ^26^ Studies across multiple therapeutic areas have reviewed this lack of racial/ethnic diversity and have identified which psychosocial and cultural attributes act as barriers and motivators to study participation.^7^, ^27^, ^28^, ^29^, ^30^, ^31^, ^32^, ^33^ For example, having a lower income, poor education or a lack of research study resources in a native, culturally appropriate language may be barriers to participation.^30^, ^34^ This study sought to understand what other factors may exist to motivate or prevent someone from participating in research.

There is a growing focus on community‐based participatory research (CBPR) to achieve diversity in the study population.^35^ CBPR follows the bottom‐up approach (starting with community members to identify salient issues important to a particular population) instead of the traditional top‐down approach (where external organizations identify an agenda that may not be reflective of the needs of a community). This approach maximizes community participation and patient retention as it considers community members as invested partners in the intervention and outcomes.^36^, ^37^ Research supports that the CBPR approach is effective in recruiting, retaining and improving behavioural and health‐related outcomes in disadvantaged communities.^38^, ^39^, ^40^, ^41^

US Food and Drug Administration (FDA) recommends a patient‐focused drug development approach to ensure that the views, needs, preferences and interactions of patients are captured and meaningfully integrated throughout the lifecycle of a medical product.^42^ Although it is crucial to embrace patient‐focused research, the existing literature reveals an unmet need in this area.^33^, ^43^ Qualitative research provides an insight into the experiences of participants and enables researchers to understand rich explanations and descriptions in local contexts.^44^ This type of research can help capture underrepresented patient experiences and may enhance the engagement of diverse patient groups in clinical research.^45^ In this study, focus groups with a diverse group of patients across multiple disease areas were conducted to better understand potential motivators and barriers to study participation across different races and ethnic groups. This study also assessed the preferred delivery of education and information to support healthcare decision‐making and the role of the community.

## 2 METHODS

### 2.1 Overview of Patient Engagement Research Council (PERC) model and study participants

The present study gathered and analysed information from focus groups comprising 26 patients currently participating in the PERC program of the sponsor.

The PERC program constitutes a diverse group of participants who suffer from chronic disease, are self‐aware of their condition and provide their input through a structured series of specific research activities. Participants were recruited through outreach to patient advocacy organizations, online advertising websites, social media, and physician referrals. Recruitment targeted ‘everyday’ participants with a variety of healthcare experiences; some participants were very involved in healthcare decision‐making, others less. Participants were not exclusively experts or advocates in their disease area. The screening and recruitment process for this study allowed participants to self‐identify race and ethnicity, and these identifiers were taken into account when designing and analyzing research. Subjective sampling was used, and demographics such as race/ethnicity were often prioritized in the recruitment process to ensure adequate representation. Individuals were subjected to a confidential and thorough screening and interview process before selection by a series of questions, assessing prior participation in any research study, self‐identification in terms of race/ethnicity, and the highest level of education received. Of patients who expressed interest, on average, approximately 64% were interviewed; 57% were eligible to participate and 42% were invited to become PERC participants. Council members were compensated for their time participating in research activities and each research opportunity was voluntary.

At the time of this study, the PERC program consisted of 108 participants, 30% of which self‐identified as African American/Black, 8% as Hispanic/Latino and 7% as Asian American.

### 2.2 Procedures and study groups

Research questions were developed to address the key study objectives before conducting the focus groups. The questions were formulated by a research specialist who possessed experience in culturally appropriate research methods and patient literacy and were structured around validated health behaviour principles that were used by the research team across similar studies. A discussion guide was developed by a research specialist with additional review by senior researchers and sponsor representatives. Participants involved in these sessions had one of the following self‐reported disease conditions: peripheral arterial disease, venous thromboembolism, cardiovascular disease, inflammatory bowel disease, ankylosing spondylitis, psoriatic arthritis, prostate cancer, multiple sclerosis or pulmonary arterial hypertension. Information from five focus groups was gathered and analysed; each discussion was conducted for 90 min on different dates.

In total, 26 participants [males: *n* = 11 (42.3%); females: *n* = 15 (57.7%)] engaged in this study. Participants were grouped by self‐identified race/ethnicity to facilitate open discussion; these included two African American/Black groups [*n* = 11 (42.3%)], one Hispanic/Latino group [*n* = 5 (19.2%)], one Asian American group [*n* = 4 (15.4%)] and one white group [*n* = 6 (23.1%)] (see Table 1). Nine participants had previous experience participating in clinical research.

**Table 1:** Demographics of  the participants

Demographics (total participants, *N* = 26)	
Gender (*n*)	
Male	11
Female	15
Ethnicity (*n*)	
African American/Black	11
Hispanic/Latino	5
Asian American	4
white	6
Participants in different age groups (*n*)	
18–34 years	6
35–44 years	6
44–54 years	4
55 years and older	10
Education level (%)	
Less than high school	2.8
High school	2.8
Some college	4.15
Trade school	1.4
Associate's degree	1.4
Bachelor's degree	23
Post‐graduate	38
Disease areas (*n*)	
Peripheral arterial disease	3
Venous thromboembolism	5
Cardiovascular disease	1
Inflammatory bowel disease	6
Ankylosing spondylitis	1
Psoriatic arthritis	2
Prostate cancer	2
Multiple sclerosis	3
Pulmonary arterial hypertension	3

Participants were informed that no treatments would be provided, and they could withdraw at any time. Additionally, a consent and release form was signed by the participants that communicated confidentiality and Health Insurance Portability and Accountability Act (HIPAA)‐compliant practices. All data were deidentified; thus, no ethics board review was required. The purpose of this study was to collect personal perspectives and qualitative insights from the participants. The study was also conducted in accordance with the Helsinki Declaration of 1964 and its later amendments. All sessions were conducted virtually, and participants joined from their homes.

**Figure 1:**
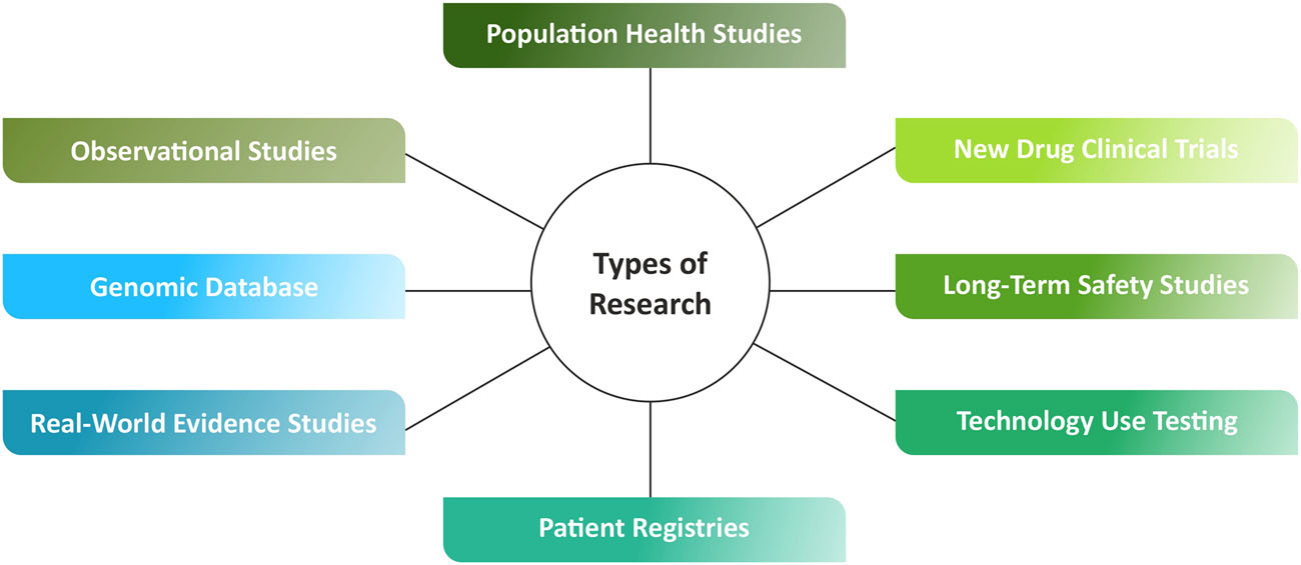
Types of clinical research conducted by pharmaceutical companies, academic institutions, non‐profits, citizen scientists and government agencies.

For each focus group, participants were first introduced to the types of clinical research to provide the foundation for discussion and the basic role of the participant (Figure 1). Participants were then asked to share their experiences and opinions regarding participation in research studies, factors that they considered as motivators or barriers to study participation, and sources of information with a focus on understanding the factors enhancing trustworthiness.

### 2.3 Data management and analysis

All discussions lasted 2 h each and were audio‐recorded and transcribed. Following the sessions, recordings for transcription were submitted through a transcription firm experienced in transcribing medical market research.^46^ The research team utilized conceptualization and conversational (narrative) analysis to identify concepts in the data from this study. A senior research specialist with experience in narrative analysis and drawing insights from interactions with diverse populations directly observed the data (during data collection, and subsequently, through transcript analysis) and identified concepts, with support from a research associate. Using an iterative process, analytic insights were tested against new observations; concepts were refined as those continued to emerge from the data. This process continued until all the data were analysed and the frequency of each concept had been examined to understand its importance. The resultant concepts were then reviewed by additional senior researchers who attended the focus groups. The scope of this project did not include formal thematic coding of the qualitative data.

## 3 RESULTS

### 3.1 Motivators to study participation

Motivators to study participation cited during the discussions included access to novel treatments for participants who were ‘out of options’ for their diseases, altruism, better or free medical coverage, compensation, curiosity to learn more about their disease and improved medical attention. A few felt a responsibility to represent an under‐represented group, be it their gender identity (female), sexual orientation (LGBTQ+), race (African American/Black) or even geographic location (rural). In particular, African American/Black participants suggested that medications are not typically designed with them in mind, so involvement in research studies was perceived as a positive change to this pattern of underrepresentation. Each group identified better or free coverage as an advantage, especially among those who struggled to afford expensive care/treatment. A few participants expressed willingness to enroll in research studies simply on the strength of their healthcare professional's (HCP's) recommendation (see Box 1 for selected quotes stating motivators from participants). Many participants indicated that they would trust their ‘gut instinct’, or seek out a personal referral from a trusted source of information. African American/Black respondents were more likely to trust and seek care from other providers of their same race/ethnicity (see Box 2 for selected quotes reflecting the perceived impact of race and ethnicity on access to care).

Box 1 **Sample quotes from participants stating motivators for study participation**

I participate because I am a female, and a lot [of research] is not geared to the female…or the African American.

–African American/Black

I have an extremely rare disease…and live in a rural area…nobody around here could even treat it…but I've been lucky to have treatment and travel covered.

–white

Lack of insurance access…good doctor access…good consistent care.

–Asian American

If my doctor said we want to try something new, I'd jump on it. And is it even possible to say that you're just curious?

–Hispanic/Latino

Participating in clinical trials allows you to learn more [about your condition] …and colonoscopies would be free.

–African American/Black

Box 2: **Sample quotes from participants reflecting the perceived impact of race and ethnicity on access to care**

A male African American doctor, I feel he relates to me and my issues better…African American more than male.

–African American/Black

Sometimes physicians are shocked that someone who is African American would be interested [in participating], so they may not even mention it to us…I feel like even my diagnosis was delayed because of my race…and then my family [gets defensive and suggests] that they're going to be experimenting on me.

*–*African American/Black

… Generally speaking, white men tend to make me a little nervous because I feel spoken down to at times.

*–*Hispanic/Latino

If the doctor were a Christian or not, it would have absolutely no effect on me.

*–*Asian American

I get the information, do my own research to compare, I synthesize it, and then pray on it.

I trust God with the final decision. And my wife, of course!

*–*African American/Black

### 3.2 Barriers to study participation

In our discussion, fear was found to be the primary obstacle to research study involvement. Specifically, participants feared: side effects (short‐term or long‐term) due to untested medications; potential loss of standard of care (SOC) or other treatment disruptions; violating cultural norms, including defying family members; stigma; their data being tracked (e.g., registries); misinformation; not being able to comprehend the salient points of a trial and/or research study because of health literacy or language barriers; and being subject to abuse based on historical events (see Box 3). While abuse based on historical events was cited mostly in the African American/Black group, a white female of orthodox Jewish heritage suggested that her community members are wary of experimental treatments due to warnings from Holocaust survivors. Historical abuse contributed to mistrust and hesitancy to be the first to try a treatment and avoidance of participation in clinical research. No respondents in the Hispanic/Latino or the Asian American group referred to these events, although one Latino male alluded to neglect due to his LGBTQ+ affiliation. Participants indicated difficulties understanding US FDA and institutional review board (IRB) oversight which may impact willingness to participate in research. Logistical issues were generally not cited as obstacles.

Box 3: **Sample quotes from participants stating fears and potential barriers to study participation**

When it comes to taking something that's not on the market or FDA approved, you're obviously going to be fearful.

–African American/Black

Misinformation…especially when it comes to minority populations…and I've seen clinical trial teams and coordinators be very stigmatizing towards low/moderate income communities.

–African American/Black

Anything that changes my medication routine now would be an obstacle for me…also the language barrier and complexity of health care [for Latino immigrants].

–Hispanic/Latino

It becomes a balance of the inconvenience versus the possible benefit…I'd want to make sure I was doing something that wouldn't have a permanent [negative] impact, like if I was given the placebo.

–white

People in my Orthodox community are very hesitant to be involved…whenever people in my community find out that I am in a study, they think I am absolutely insane…because a lot of medical treatment was done on Jews during the Holocaust…so you never want to do anything first…only if the general American public accepts it first.

–white

I need a dictionary to understand the consent forms in order to get the information!

–African American/Black

Additional barriers identified included a lack of trust in the overall study process and in pharmaceutical companies. While some participants were willing to acknowledge the contribution of pharmaceutical companies in advancing diversity and equity in research, they displayed hesitation in considering them a trusted source. Affiliation with an advocacy organization, or an endorsement by a member of their identified community, however, was perceived to be an effective way to enhance the trustworthiness of a pharmaceutical company. Overall, there was reluctance to trust any entity or institution with a financial stake in the outcome of the study.

### 3.3 Preferred delivery of education and information to support healthcare decision‐making and the role of the community

Participants were less likely to consult primary care physicians about disease‐specific questions and tend to address these issues with their specialists instead. Although many viewed their relationship with their doctors as a partnership, they also liked to self‐educate about their specific health condition(s) and elicit medical opinions on information obtained rather than relying exclusively on their doctor as a source. Preferred sources for educating oneself included: Internet searches, literature reviews, eliciting opinions from educated friends/family and exposure to experiences of other participants. WebMD was considered too elementary to be viewed as a credible source of information; websites with extensive scientific content, such as NIH, Mayo Clinic, Johns Hopkins and PubMed were the preferred choices.

Trust in social media for medical guidance was roughly predicated on the age of the participant, and to a lesser extent, the platform. In general, younger participants (age: mean 48.7 years; median 46 years) trusted social media. Twitter and Reddit/sub Reddits were cited specifically, and Facebook appears to be the least trusted of all platforms. Few participants also preferred utilizing Google searches, YouTube and other social media platforms. Connecting with peers living with the same condition appeared to be extremely important, especially among younger participants.

Religion was found to play an important part in medical decisions; however, its role appeared to vary for different ethnic groups. Association with religious groups was the strongest among African American/Black and orthodox Jewish white respondents. African American/Black participants cited ‘prayer’ as important, after arming themselves with as much knowledge as possible regarding a potential research study. For other ethnic groups, the religious social group was a more significant influencer of medical decisions than the religious beliefs themselves. For example, an Asian American respondent stated they consulted the medical professionals from their church community on medical decisions. A few selected quotes from participants on the preferred delivery of education and information to support healthcare decision‐making and the role of the community are available in Box 4.

Box 4: **Sample quotes from participants on the preferred delivery of education and information to support healthcare decision‐making and the role of the community**

My GP is great, but I would not let them make decisions about my VTE…And I find my female doctors are more focused on my particular needs.

*–*white

Showing up authentically is the most vitally important thing…and the main component is building trust…build partnerships within the communities…the community rapport is what you have to build on.

*–*African American/Black

The role of social media and whatever health community you are a part of is really crucial…a lot of physicians have taken an active role in their community –not so much Facebook, but definitely Twitter…I think it's a very big part of where a lot patients get their information from.

*–*white

I have a great community of other patients I met over the years at various psoriasis conferences…they are my go‐to for emotional support…and there's always quite a bit of information running around that group.

*–*Asian American

I like to talk to other community members—a social media community that I built around IBD…plus a lot of Google searches, YouTube…and emailing back and forth with my doctor.

*–*Hispanic/Latino

My community consists of my (familial) medical team. And a group called Black Health Matters…they help you understand the risk versus the benefit [of trial participation].

*–*African America/Black

I would identify my community as the LGBTQ+ community…and the chronic illness/disabled community. I am very active on Twitter, so I have connected with others even if we don't share a diagnosis.

*–*Hispanic/Latino

My community is my Reddit group—Peeps with UC and Crohn's. I definitely trust their opinions.

*–*African American/Black

I'm part of 2 communities, being in the healthcare field. But being part of the PH community is huge…My healthcare side helps me to investigate what I hear in the PH community.

*–*Asian American

Overall, participants desired transparency and engagement to build trust and continued partnership to enhance healthcare decision‐making. Participants felt ‘showing up authentically’ and building relationships at the community level—in both real‐world and virtual settings—are important considerations for researchers. While a few participants decided by themselves, others preferred to consult individuals they trust; seeking information from communities they relate to, such as LGBTQ+ and disease‐specific support groups.

## 4 DISCUSSION

This study underscores the importance of engaging with patients directly to understand their individual perspectives and experiences to enhance participation in research studies. The findings highlight important motivators and barriers for research study participation among underrepresented racial and ethnic groups, and the significance of social identity, trusted sources and community engagement in healthcare decision‐making. It is critical for pharmaceutical companies and other entities conducting research studies to build trust and continued partnerships with participants through transparency and direct engagement with patients in their own communities.

Our study detected several racial and ethnic differences. Perceptions among the African American/Black groups in our study regarding healthcare and research participation differed from other groups, especially displaying increased trust for HCPs who share their race/ethnicities. This insight should steer research sponsors to increase the involvement of HCPs across racial and ethnic groups in research and to partner with religious/cultural community groups to enhance trust among prospective study participants. An example from the literature highlights the usefulness of CBPR, through which community‐level engagements are carried out to improve diversity and inclusion in clinical research.^35^, ^40^, ^41^, ^47^ The findings of this study support the need for pharmaceutical companies and research investigators to apply CBPR or similar models when planning and designing clinical research studies.

Our findings on motivators and barriers align with those of previously published studies reporting factors that contribute to the lack of racial/ethnic diversity in study participation.^7^, ^29^, ^30^, ^31^, ^32^, ^33^ The key barriers identified in previously published research include mistrust in pharmaceutical companies sponsoring trials, as well as the scientific and medical community at large, fear, concerns/discomfort with the research process, burden, time and resource constraints, and lack of awareness about the importance of research studies. In addition to this, cost concerns or health insurance, demographic factors and cultural factors (e.g., lack of culturally pertinent education about research studies, use of native language for target populations and communication) were also reported.^29^, ^30^, ^32^, ^33^ Facilitators included positive provider–patient relationships, enthusiastic physicians with effective communication skills and feelings of altruism.^32^ Educational strategies and transparent communication between participants and physicians/clinical research staff could encourage study participation.^29^ Wider participation from various racial, ethnic and cultural backgrounds could also be achieved by partnering with patient advocacy organizations and local community organizations and engaging partners through websites, social media and applications.^7^, ^33^

Significantly, when discussing fears and barriers to participating in clinical research, participants shared the importance of self‐identification beyond race/ethnicity. For example, participants shared that gender identity or sexual preference, and religious affiliation could influence their willingness to participate in a research study. This complexity signals the importance of considering an individual's influencing ‘ecosystem’ and the concept of ‘intersectionality’ when attempting to engage populations in research. It is important to realize self‐identity not only as belonging to a race but also multiple other traits (such as gender, geography, etc.). This complexity should be acknowledged and accounted for in research.^48^ Further understanding of how patient multidimensionality impacts decisions to seek out and participate in research, and recommendations to address, are an opportunity for future exploration.

Mistrust towards clinical research and the pharmaceutical industry is a challenge; it is attributed to historical experiences, education levels, language and ethnic backgrounds,^49^, ^50^ as justified by examples in the past.^51^, ^52^ Participants identified the need for sponsors to engage at the community level to build trust and support educational efforts related to the importance of study participation. This insight reflects the willingness of a person to participate in research if approaches focus on easing individual anxiety through community engagement.^7^ Additionally, addressing low literacy and language differences requires culturally appropriate patient education. For clinical trials, patient education should emphasize US FDA and IRB oversight with a clear explanation of exactly what an IRB is and the role it plays to protect the rights of trial participants. Additionally, specialist HCPs, identified as the most trusted sources,^7^ should be trained to ensure adequate patient education, in easy‐to‐understand language, regarding potential studies and the importance of diversity and inclusion. Educating physicians may also serve to alleviate fears identified surrounding the loss of SOC treatment, side effects and treatment disruption.^32^

This study provides valuable insights for increasing diversity and inclusion in research. While qualitative research is a critical tool to gather the patient voice, inherent limitations exist. There is a tendency for the emergence of socially acceptable opinions and bias development within the group due to the dominance effect and groupthink. Small sample sizes cause the research to be mainly exploratory in nature, as the number of participants in each group and the duration of focus group discussions do not allow testing of the data saturation point.^53^ Prior participation in research studies by some participants might indicate increased openness to clinical research, knowledge and awareness that could shape their perspectives either positively or negatively. Further, responses were not coded, which could have resulted in an interpretation bias. Therefore, an opportunity exists for future research to address these limitations around data collection and analysis. The drafting of this article was planned only after data analysation and was intended for hypothesis generation, not confirmation. In addition, the individuals who agreed to participate in this study were likely to be relatively more health‐engaged or actively aware of their disease, which could potentially limit the generalizability of these results to broader patient populations.

## 5 CONCLUSION

Our study generated significant qualitative patient perspectives regarding potential barriers to participation in research studies, highlighting racial and ethnic differences, and the role of engaging with communities to help overcome these barriers to achieve diversity and inclusion in studies. These focus groups highlight the importance of programs that foster bidirectional collaboration between pharmaceutical or other entity sponsors and community members. The findings and recommendations presented in this study could be further integrated into the research study recruitment framework and processes to support an engagement strategy, with an enhanced focus on the intersectionality of the ‘whole patient’ to build trust and partnership with patients and their communities.

## AUTHOR CONTRIBUTIONS

All authors contributed to data analysis, drafting or revising the article, gave final approval of the version to be published, and agree to be accountable for all aspects of the work.

## CONFLICTS OF INTEREST

L. S., J. P., and G. G. are employees of Janssen Scientific Affairs, LLC. V. P., C. K., and W. P. are employees of CorEvitas, LLC, which derives its profits from interactions with pharmaceutical sponsors.

## Data Availability

The data that support the findings of this study are available on request from the corresponding author. The data are not publicly available due to privacy or ethical restrictions.
